# Uncontrolled Web-Based Administration of Surveys on Factual Health-Related Knowledge: A Randomized Study of Untimed Versus Timed Quizzing

**DOI:** 10.2196/jmir.3734

**Published:** 2015-04-13

**Authors:** Alexander Domnich, Donatella Panatto, Alessio Signori, Nicola Luigi Bragazzi, Maria Luisa Cristina, Daniela Amicizia, Roberto Gasparini

**Affiliations:** ^1^Department of Health SciencesUniversity of GenoaGenoaItaly

**Keywords:** knowledge questionnaires, online survey, time limit, uncontrolled survey administration, cheating, e-cheating, cribbed answers

## Abstract

**Background:**

Health knowledge and literacy are among the main determinants of health. Assessment of these issues via Web-based surveys is growing continuously. Research has suggested that approximately one-fifth of respondents submit cribbed answers, or cheat, on factual knowledge items, which may lead to measurement error. However, little is known about methods of discouraging cheating in Web-based surveys on health knowledge.

**Objective:**

This study aimed at exploring the usefulness of imposing a survey time limit to prevent help-seeking and cheating.

**Methods:**

On the basis of sample size estimation, 94 undergraduate students were randomly assigned in a 1:1 ratio to complete a Web-based survey on nutrition knowledge, with or without a time limit of 15 minutes (30 seconds per item); the topic of nutrition was chosen because of its particular relevance to public health. The questionnaire consisted of two parts. The first was the validated consumer-oriented nutrition knowledge scale (CoNKS) consisting of 20 true/false items; the second was an ad hoc questionnaire (AHQ) containing 10 questions that would be very difficult for people without health care qualifications to answer correctly. It therefore aimed at measuring cribbing and not nutrition knowledge. AHQ items were somewhat encyclopedic and amenable to Web searching, while CoNKS items had more complex wording, so that simple copying/pasting of a question in a search string would not produce an immediate correct answer.

**Results:**

A total of 72 of the 94 subjects started the survey. Dropout rates were similar in both groups (11%, 4/35 and 14%, 5/37 in the untimed and timed groups, respectively). Most participants completed the survey from portable devices, such as mobile phones and tablets. To complete the survey, participants in the untimed group took a median 2.3 minutes longer than those in the timed group; the effect size was small (Cohen’s *r*=.29). Subjects in the untimed group scored significantly higher on CoNKS (mean difference of 1.2 points, *P*=.008) and the effect size was medium (Cohen’s d=0.67). By contrast, no significant between-group difference in AHQ scores was documented. Unexpectedly high AHQ scores were recorded in 23% (7/31) and 19% (6/32) untimed and timed respondents, respectively, very probably owing to “e-cheating”.

**Conclusions:**

Cribbing answers to health knowledge items in researcher-uncontrolled conditions is likely to lead to overestimation of people’s knowledge; this should be considered during the design and implementation of Web-based surveys. Setting a time limit alone may not completely prevent cheating, as some cheats may be very fast in Web searching. More complex and contextualized wording of items and checking for the “findability” properties of items before implementing a Web-based health knowledge survey may discourage help-seeking, thus reducing measurement error. Studies with larger sample sizes and diverse populations are needed to confirm our results.

## Introduction

Measuring people’s knowledge of health-related topics in order to assess whether they are sufficiently aware of prevention, medication, and self-care is of particular importance, as health knowledge and health literacy are among the main determinants of health behavior and health status [1,2]. Gaps in people’s knowledge, as identified through surveys, can subsequently be targeted by specific information and education interventions. In order to assess people’s knowledge of health topics, several tools have been developed in the past few years, including questionnaires, tests, and quizzes on medical/general health [3,4], disease-specific [5-8], and risk factor [9-11] knowledge. Moreover, knowledge items are an essential part of KAP (knowledge-attitude-practices) surveys [12], which are widely used in health care research.

Data collection by means of questionnaires varies in terms of how potential respondents are enrolled, the vehicle used for survey delivery, and the mode of questionnaire administration; all these factors may seriously affect the quality of the data [13]. Web-based surveys have now become important tools in epidemiological and health care data collection and their use seems destined to grow rapidly [14]. Potential advantages and shortcomings of Web-based surveys have been described extensively and reported elsewhere [14-16]. Briefly, the advantages are: the possibility to enroll subjects in distant locations and hard-to-reach and hard-to-involve populations, the automatic nature of the system, which saves researchers time and effort, and the potential cost savings. The disadvantages include uncertainty regarding the quality and validity of the data collected, the representativeness of data, due mainly to the digital divide, and general concerns about the design, implementation, and evaluation of a Web survey [15].

Like other research fields, the assessment of knowledge on health-related topics via Web-based surveys is increasing [3,17-26]. However, since surveys are completed in an environment that is beyond the researcher’s control, the Web-based administration of a knowledge questionnaire presents considerable shortcomings. First, a positive response bias is likely to be introduced, since people with more knowledge on a health topic would be more likely to fill in a Web-based survey, which may yield non-representative high scores [27]. For instance, parents with lower nutrition knowledge have been shown to have a higher dropout rate in Web-based assessments of nutrition knowledge, dietary habits, and attitudes of their children [28]. Second, as has been described by Handré et al [29], various social and asocial factors linked to unsupervised survey administration (such as interaction with other people or engagement in activities unrelated to the survey) may have a direct influence on respondents, leading to data contamination. Indeed, these authors found that, among students who were invited to complete a survey in an environment of their choice, 42% were engaged in conversation, one-third had someone present in the room, 21% surfed the Internet, 5.5% received help on questionnaires, and 3.3% changed their answers following someone else’s advice. In a recent paper, Jensen and Thomsen [30] examined the influence of nonrandom measurement error on the average level of political knowledge scores obtained in a Web survey. The authors started from the fact that, in comparison with face-to-face or telephone surveys, Web-based surveys tend to record higher knowledge scores, probably as a result of cribbed answers or “e-cheating” (which can be broadly defined as the use of information technology in any type of cheating [31]), when participants are able to interrupt survey compilation and look for correct answers via common search engines. Indeed, they found a high rate of self-reported e-cheating (22.3%) and concluded that this may be an important source of measurement error. Similarly, it has been noted that respondents more frequently give correct answers on cholesterol knowledge items online than in face-to-face interviews [32]. Another potential concern is that participants may respond carelessly owing to lack of interest, in which case their scores are likely to be too low [33,34].

The authors of some earlier studies that used Web-based surveys to assess knowledge of health-related topics through questionnaires administered in unsupervised settings have acknowledged the possibility that respondents might cheat by using additional information sources [3,18,20,25]. In these studies, attempts to prevent cheating were made by ensuring anonymity and encouraging interviewees not to use additional sources. Such attempts, however, may not be efficacious enough.

Although the problem of cheating in Web survey research is recognized, little is known about practical methods of controlling for its effects on data quality, especially in research on health topics. It has been proposed that picture-based items should be used, in order to prevent respondents from using search engines to find the correct answers [35]; such an approach, however, may be difficult to implement in health surveys. Adding specifically designed items to measure self-reported cheating and tabbed browsing should also be considered [30]; however, this strategy risks engendering a social desirability bias, whereby people may underreport engaging in socially undesirable activities [36] such as cheating. Another easy-to-implement method of mitigating the effects of cheating is recording survey completion time and imposing time limits. Malhotra [37] has suggested that survey completion time should always be included as a control variable in statistical models. Indeed, the time taken to complete a Web-based survey can seriously affect data quality [37] in two ways. On the one hand, respondents who rush through a questionnaire may provide less thoughtful [37] or careless answers [33,34]. On the other hand, a long response time may reflect social distraction [29], help-seeking [29,35], or simply greater thought. It has been suggested that setting a time limit may reduce collaboration and help-seeking [38]. The time-based approach seems to be plausible, assuming that cheaters are generally slower than non-cheaters since searching the Web requires some time [30]. However, the effectiveness of time restriction seems to be uncertain, as has been documented in social and political research. Indeed, while Strabac and Aalberg [35] claim that imposing a time limit of half a minute for each answer may solve the problem of cheating, Jensen and Thomsen [30] have demonstrated that e-cheaters are generally quick and that the mean time needed to answer a question is often below 30 seconds.

The present randomized study aimed to compare respondents’ performances on a health knowledge survey administered with or without a time limit through an uncontrolled Web-based modality. The subject of the survey was nutrition-related knowledge, a topic of great relevance to public health and one of the most widely studied by means of various data collection modalities. We first hypothesized that respondents who completed the questionnaire within a limited time would score fewer points than those working without a time limit, as they would have less time for help-seeking, social interaction, and e-cheating. Our second hypothesis was that the differences in quiz performance between time-restricted and time-unrestricted groups depend on the type of questionnaire—it would be greater in quizzes more amenable to cheating (primarily as a result of good Web “findability” properties of items). The study did not aim to assess factual nutrition-related knowledge; rather, it constitutes a further step toward finding an optimal modality of conducting Web-based health-related surveys and reducing measurement error.

## Methods

### Study Setting, Participants, and Procedure

A convenience sample of approximately 150 third-year students at Genoa University (faculties of architecture and education sciences, about 65% females) were recruited for the study in May 2014. The students were told that the aim of the study was to test the feasibility of the Web-based administration of a nutrition-related questionnaire. A short description of the study, the survey instrument, and the modality of survey completion were provided by a researcher from outside the faculty. Students were informed that participation was voluntary, that anonymity was guaranteed, and that the researchers would not know who had filled out the survey. No incentives to participate in the survey were offered. After presentation of the study, the email addresses of those who agreed to participate were collected; all volunteers were able to connect to the Web.

On the day of recruitment, students were randomly allocated into two groups on a 1:1 basis by computer-generated randomization to fill in the questionnaire either without a time limit (TL–) or with a time limit (TL+). Participants were then sent an email containing brief instructions and a direct link to the surveys.

Ethical approval for this anonymous survey was not deemed necessary, since its nature was non-medical and non-interventional; no sensitive data or personal information were collected from volunteers.

### Survey Instrument and Outcome Measures

The test consisted of two main parts plus two items on sex and age. There was no “don’t know” option, but participants were instructed that they were free to leave out any item if they did not know/were not sure of the correct answer. Only the two items on age and sex were obligatory, as was clearly indicated (by an asterisk). The first part was the validated consumer-oriented nutrition knowledge scale (CoNKS) [11] consisting of 20 true/false items. This tool was chosen because of its brevity, good internal reliability, and criterion and construct validity [11]. CoNKS was translated from English to Italian by means of a back-translation technique by two professional translators. The second part was an ad hoc questionnaire (AHQ) comprising 10 multiple-choice items with 5 response options, one of which was correct (see Multimedia Appendix 1). These 10 items were intentionally designed to be difficult for people without a health care/nutrition background to answer correctly; indeed, they were not intended to measure functional nutrition knowledge, but as a proxy measurement of participants’ cheating [39]. At the same time, the AHQ items had good “findability” properties as a result of their “encyclopedic” nature, thus enabling the correct answers to be found easily on the Web. Each of the 10 items was pretested by copying a question into the Google search string; the correct answer could be found directly on the screen among the first 10 search results.

Each correct answer was scored as 1, while incorrect or missing answers were scored as 0. In sum, the resulting total CoNKS score could vary from 0 to 20, while AHQ yielded an overall score out of 10.

In a preliminary study, we had administered the AHQ to 21-23 year old students of engineering (n=15) in an in-class paper-and-pencil researcher-controlled setting. The mean score was 2.3 (SD 1.0) with a range from 1 to 4 points. We therefore assumed that an individual AHQ score of ≥5 in unproctored conditions would have been due to e-cheating.

The survey was implemented by means of professional Web-based survey software QuestionPro. In order to increase respondents’ motivation, the layout of both surveys was endowed with a clearly visible progress indicator and all items were scrollable and skippable [40]. Furthermore, this professional software was chosen because of its automatic optimization, such as larger text and easy-to-use buttons, during completion from mobile devices. The layout of both the TL- and TL+ questionnaires was identical, the only difference between the two being the presence of a time limit in the latter case. The time limit of 15 minutes (approximately 30 seconds per question) was imposed in light of previous research [35] and following agreement among research team members that this limit was reasonable. A countdown timer situated next to the progress indicator was clearly visible at the top of the screen. The time taken to complete the survey was automatically recorded by the software. Before clicking on the link, subjects did not know whether or not they would have a time limit to complete the quiz.

The rates of responses and dropouts on both surveys were recorded. The response rate was defined as the number of subjects who started the survey as a proportion of the total number of emails sent (assuming that all emails were read), while the dropout rate was the proportion of subjects who started the survey but did not submit it. Since the QuestionPro software allows multiple entries by the same user to be identified, all submissions were screened for this eventuality. If any subject made multiple entries, all his/her entries were removed from the analysis.

Both links were active for 2 weeks. No reminders were sent, since we were unaware of which students had completed the survey and which had not.

### Statistical Analysis

Sample size was computed by means of a two-sided two-sample *t* test. As Dickson-Spillmann et al [11] found a mean difference in CoNKS scores of 1.9 points between subjects with health care or nutrition qualifications and subjects without such qualifications, we supposed that a mean difference of 2 points would have a practical significance (ie, cheating). Therefore, to detect a 2-point difference in mean CoNKS scores between the two groups, with a common SD of 3 points when power is .8 and alpha is .05, at least 36 subjects per group were needed. Considering a non-response rate of 30%, we aimed to enroll 94 subjects.

For descriptive purposes, quantitative variables were expressed as means with SDs or medians with ranges. Descriptive data were expressed as frequencies and percentages with 95% confidence intervals (CIs). To compare categorical data, the *χ* ^2^test with Yates correction or Fisher’s exact test (in case ≥20% of expected frequencies were <5) were performed. To compare between-group differences in continuous variables, the two-sample Student’s *t* test was used when data in each sample showed approximately normal distribution, which was preliminarily assessed by means of both visual inspection and Shapiro-Wilk test; otherwise, the Mann-Whitney *U* test was preferred. The effect size for normally distributed data was measured by means of Cohen’s *d* with 95% CIs and *U*_*3*_ index. Cohen’s *d* was interpreted as: small (*d*=0.2), medium (*d*=0.5), and large (*d*=0.8). The effect size for the Mann-Whitney test was expressed as Cohen’s *r*=*z*/√*n* and interpreted as follows: small (*r*=0.1), medium (*r*=0.3), and large (*r*=0.5) [41].

To assess whether the impact of time limit group (TL− or TL+) was similar in CoNKS and AHQ, a linear mixed model with interaction between the type of quiz and group was used. Since the two scores were on different scales, the *z*-score transformation was applied first. The linear mixed model enabled us to account for possible correlation between scores obtained by the same subject.

Statistical significance was set at a two-sided *P* value of <.05. All data were analyzed by means of the R stats package, version 3.0.1 [42].

## Results

### Descriptive Statistics

Of 107 volunteers, 94 (47 per group) were randomized to receive the link to either TL– or TL+. No multiple entries were registered; both surveys were started by 72 individual visitors. Response rates were similar in both groups (75%, 35/47; 95% CI 61-85% and 79%, 37/47; 95% CI 65-89% in TL– and TL+ groups, respectively; *χ* ^2^_1_=0.06, *P*=.81). The between-group dropout rate did not differ (*P*=.99, Fisher’s exact test): 4 participants in the TL– group (11%, 4/35) and 5 in the TL+ group (14%, 5/37) dropped out before submitting responses. Among the 9 dropouts, 7 (78%; 95% CI 44-96%) looked at the survey items without answering any question, while the remaining 2 dropped out after answering some items. Thus, 63 complete surveys (31 and 32 in TL– and TL+ groups, respectively) were analyzed. Overall, both groups were comparable in terms of age, sex, and device used to complete the survey. Notably, 60% (43/72; 95% CI 48-71%) of respondents completed the survey from portable devices (Table 1).

**Table 1:** Sex, age, and devices used by study participants.

Parameter		TL– (with no time limit)	TL+ (with time limit)	Statistical test
Sex, female^a^, n (%; 95% CI)		25/31 (81; 64-92)	26/32 (81; 65-92)	*χ* ^2^_1_=0.07, *P*=.80
**Age, years^a^**				*t*_61_=1.44, *P*=.16
	mean (SD)	22.2 (1.8)	21.6 (1.7)	
	median (range)	22.0 (20-27)	21.0 (19-26)	
**Device used** **n (%; 95% CI)** ^b^				Fisher’s exact test, *P*=.94
	Desktop/laptop	15/35 (43; 27-60)	14/37 (38; 23-54)	
	Mobile phone	16/35 (46; 30-62)	19/37 (51; 36-67)	
	Tablet	4/35 (11; 4-25)	4/37 (11; 4-24)	

^a^Based on subjects who completed the survey.

^b^Based on subjects who started the survey, since only overall statistics were available.

### Outcome Measures

The distribution of survey completion time was substantially right-skewed in both groups (skewness coefficients of 1.6 and 2.0 in TL– and TL+ groups, respectively). TL– group respondents spent more time completing the survey than TL+ group respondents (median 7.8 minutes, range 2.9-30.5 minutes vs median 5.5 minutes, range 3.4-14.7 minutes); the Mann-Whitney test showed a significant difference (*z*_*U*_=2.30, *P*=.021). The effect size was, however, judged small (*r*=0.29).

As shown in Table 2, subjects in the TL– group scored significantly higher on CoNKS than those in the TL+ group; Cohen’s *d* was medium, being 0.67 (95% CI 0.54-0.80); as shown by the *U*_*3*_ index, 75% of the TL– group scored above the mean of the TL+ group. By contrast, no statistically significant difference emerged in mean AHQ scores or in the percentage with a score of ≥5. The proportion of non-response items tended to be higher in the TL+ group, especially on CoNKS; the difference, however, did not reach the alpha level of 5% (Table 2).

In the linear mixed model, the group effect (TL– and TL+) on the standardized global score derived from the two questionnaires was statistically significant (*P*=.020), while the interaction between type of questionnaire and group was not (*P*=.19).

**Table 2:** Participants’ performances on the survey instruments.

Questionnaire	Parameter	TL– (with no time limit)	TL+ (with time limit)	Statistical test
**CoNKS (consumer-oriented nutrition knowledge scale)**				
	Total score, mean (SD)	16.5 (1.9)	15.3 (1.7)	*t*_61_=2.73, *P*=.008
	median (range)	17.0 (11-20)	15.5 (12-18)	
	Non-response items, n (%; 95% CI)	1/620 (0.2; 0-0.8)	6/640 (0.9; 0-2)	Fisher’s exact test, *P*=.13
**AHQ (ad hoc questionnaire)**				
	Total score, mean (SD)	3.1 (2.6)	2.6 (1.9)	*t*_61_=0.89, *P*=.38
	median (range)	3.0 (0-10)	2.5 (0-7)	
	Score ≥5,n (%; 95% CI)	7/31 (22.6; 11-40)	6/32 (18.8; 8-35)	*χ* ^2^_1_=0, *P*=.95
	Non-response items, n (%; 95% CI)	23/310 (7.4; 5-11)	29/320 (9.1; 6-13)	*χ* ^2^_1_=0.37, *P*=.55

## Discussion

### Key Contributions and Comparison With Prior Work

The present paper contributes to the existing methodological literature on Web-based data collection in epidemiological and health care research in several ways. First of all, the results of our study confirm that the risk of social interactions or e-cheating is likely in Web-based health knowledge surveys since a high proportion of respondents scored unexpectedly high on both quizzes, and this fact must be taken into account during the design and implementation of such studies. Asynchronous communication in time and space and the absence of researcher supervision in Web-based surveys make it extremely difficult to control for cribbed answers in an objective way. We intentionally did not ask participants whether they had used additional information sources or not, since cheating would very probably have been underestimated owing to the social desirability bias; however, we adopted a proxy measure of cheating described in psychological research [39]. We found substantially higher mean CoNKS scores than those recorded by Dickson-Spillmann et al [11] (difference of at least 2.3 points) during CoNKS validation. This superior performance may be partially explained by the relatively high educational level of our participants and the higher proportion of females and the young in our sample; indeed, all these factors have been found to be associated with higher CoNKS scores [11]. However, these sample characteristics can hardly explain the fact that participants in both the TL- and TL+ groups scored even higher than people with health-related or nutrition qualifications in the original study by Dickson-Spillmann et al [11] (16.5/15.3 vs 14.6). Moreover, approximately one-fifth of responders in each group scored unexpectedly high on AHQ, which is very likely due to e-cheating; a similar proportion of e-cheating had been found earlier [30].

Second, our study indicates that using timed surveys in Web-based researcher-uncontrolled assessment of knowledge of health-related topics can mitigate measurement error, as we were able to establish a significant effect of the time limit group on the quiz performance, especially on CoNKS, making our first hypothesis plausible. This finding is also of a certain practical significance, as is shown by the effect size. On the other hand, since the sample size was calculated according to the validated CoNKS survey, the non-significant between-group difference in AHQ scores was likely due to a low statistical power, although participants in the TL+ group tended to score lower. In any case, setting a time limit reduces the median time of survey completion, which, at least virtually, reduces the probability of engaging in survey-unrelated activities and help-seeking. However, imposing a time limit alone is unlikely to prevent help-seeking and e-cheating completely, since some cheaters may be particularly fast in Web searching. Indeed, Jensen and Thomsen [30] have shown that the mean response time on a knowledge item is less than 30 seconds, although the time taken by online respondents in their study varied greatly (relative SDs ranging from 144% to 1243%). High variability in response time is probably due to differences in subjects’ Web searching skills. Indeed, subjects in their late teens and twenties have been shown to be very skillful in finding given online contents relatively quickly [43]; this may explain the considerably lower variability in response time among the young adults in our sample.

Third, we suggest that the wording of questions plays an important role in terms of “findability” properties. It may therefore be useful to check whether an item can be easily Googled before undertaking a survey and, if so, to reformulate the question. Indeed, most CoNKS items have “knottier” wording than our AHQ items, and therefore have poorer “findability” properties. For instance, to locate a Web page with the correct information on CoNKS items 4 or 18 (“A healthy meal should consist of half meat, a quarter vegetables, and a quarter side dishes” and “For healthy nutrition, dairy products should be consumed in the same amounts as fruit and vegetables”), a respondent would first have to reflect on a query formulation and then scroll search results, rather than simply copying/pasting a question. To crib correct answers to these types of questions quickly, it is much more advantageous to narrow down the search results by applying an advanced search strategy. However, as demonstrated by Ivanitskaya et al [44], only one-third of students in health sciences are able to use Boolean operators and perform an advanced search. We believe that most e-cheaters in our TL+ group were not able to apply an advanced search strategy and, being under time restriction, scored lower than those in the TL- group. Conversely, as the structure of our AHQ items made them easy to Google (see Methods section), e-cheaters in both groups could find the correct answers relatively quickly, which may explain the absence of a statistically significant between-group difference in AHQ scores. Therefore, quizzes of this type, that is, of a scholastic and encyclopedic character, may be less efficacious in preventing cribbing even if a time limit is set; this finding is in line with that of Jensen and Thomsen [30]. Taken together, these facts may help to understand why we failed to confirm our second hypothesis.

It should be stressed that, if a time limit is set on a knowledge questionnaire, the limit per item or per whole questionnaire should be determined ad hoc, as the probability of e-cheating needs to be balanced against the time needed for cognitive processing. On the one hand, an ample time limit may favor e-cheaters with limited information retrieval capabilities, while on the other hand, as suggested by Jensen and Thomsen [30], it may punish relatively slow non-cheaters; in both cases, the risk of measurement error is high. We suggest that the “30 second condition” [30] per item should not be regarded as a gold standard, as several variables (eg, sociodemographic characteristics of the target population, text readability, survey layout, etc.) may interfere. To identify a more appropriate time limit, pilot researcher-supervised studies would be helpful.

Fourth, more than half of our respondents completed the surveys from portable devices such as mobile phones or tablets. It has been shown that there is little difference between computer and mobile phone administration modes, and survey outcomes assessed by the two modes are generally comparable [45,46], even though a shorter response time from mobile phones than from computers has also been observed [46]. Indeed, the median response time in our survey was substantially below the expected 15 minutes. According to Buskirk and Andrus [46], this finding may be explained by Fitts’s law of human motion, according to which selecting answers by touching the screen of a portable device is quicker than by clicking a mouse (as a result of a shorter distance from the starting point to the target). On the other hand, copying/pasting of a phrase from a touchscreen device is a little more difficult and requires a certain level of manual dexterity, and thus may discourage cheating. We believe that, in our study, these two possible explanations should be seen as complementary rather than mutually exclusive. Moreover, the mobile nature of portable devices allows their owners to complete a survey in various environments; thus, social interaction with other people may also take place, giving rise to another source of measurement error [29,47].

Fifth, the dropout rate in our study (13%, 9/72) was less than half of the 30% rate usually observed in Web-based surveys [48]. This confirms the results of previous research [40,48,49], which indicate that the structure of the survey (eg, a small number of items, absence of grids and graphically complex questions, progress indicator, scrollable and skippable items) can effectively reduce the non-participation bias in population-based studies. By contrast, time restriction is unlikely to interfere with response behavior. Three-quarters of dropouts were “lurkers”, that is, people who view the survey but do not respond to any item [49]. According to Bosnjak and Tuten [49], lurkers are generally motivated to view the survey but not to answer, probably on account of technical difficulties or loss of interest during the survey. It may plausibly be speculated that, in Web-based knowledge surveys, lurkers may contribute to a positive response bias, and therefore to the measurement error. Fortunately, modern software for conducting Web-based surveys enables us to distinguish among various types of non-respondents [49] and thus to better analyze participation patterns.

### Limitations

This study probably suffers from participation bias and positive response bias, since many more females than males completed the survey; indeed, not only were more females recruited, but also the participation rate of male students was lower than expected. It has been ascertained that women display higher participation rates in scientific studies [50] and that their participation always tends to be greater in surveys on nutrition knowledge [22-24,26]. However, since the aim of our study was not to evaluate knowledge but to compare the two survey mode conditions, these types of bias have a limited influence on the results. Moreover, application of the simple randomization procedure yielded two comparable groups.

The probability of “lucky guessing”, especially on dichotomous true/false items, should also be acknowledged. While the inclusion of “don’t know” options may discourage guessing [51], Parmenter and Wardle [52] have suggested that such options in nutrition knowledge questionnaires also constitute potential drawbacks, specifically: (1) some respondents who know the correct option but are not confident that it is correct may choose the “don’t know” response, and (2) other subjects, who could work out the correct option if they devoted a little thought to it, might mark “don’t know” as an easier alternative [52]. This is why we decided not to include an explicit “no opinion” response category; however, we tried to discourage guessing by allowing the possibility of leaving questions out.

### Conclusions

Cribbed answers to health knowledge items in researcher-unsupervised circumstances are likely. This should be considered during the design and implementation of health knowledge, health literacy, and KAP surveys, and also when comparing results from Web-based questionnaires with those obtained from proctored studies. Subsequent erroneous conclusions and overestimation of health knowledge and health literacy may contribute to poorer health outcomes [53,54]. To date, the only way to prevent cheating is to conduct a face-to-face interview [30] or a pencil-and-paper survey under strict researcher control. However, this may be more time- and resource-consuming than cyberspace surveys [15,32]. The continuous growth of technology use will probably enable novel forms of survey administration. A particularly attractive mode may be the use of synchronous Web-based and voice/video-over-Internet services such as Skype, which enable camera-to-camera video interviews to be conducted [55], thereby preventing cribbing and other forms of cheating. Although research suggests that Skype does not yet have the necessary feasibility characteristics to be used in epidemiological data collection [56], we believe it can already be used in studies targeting the young, since they are the first to have “grown up online” and are quick to adapt to novel technologies [57]. Here, we showed that imposing a time limit may only partially prevent help-seeking and further research is required in order to find an optimal cheating-sensitive vehicle for Internet-based surveys.

## Multimedia Appendix 1

Ad hoc questionnaire.

## Abbreviations

AHQ: ad hoc questionnaire
CI: confidence interval
CoNKS: consumer-oriented nutrition knowledge scale
KAP: knowledge-attitude-practices
TL–: without time limit
TL+: with time limit
