# Supplementary material for: Uncontrolled Web-Based Administration of Surveys on Factual Health-Related Knowledge: A Randomized Study of Untimed Versus Timed Quizzing
Source: J Med Internet Res. 2015 Apr 13;17(4):e94. doi: 10.2196/jmir.3734 (PMC4411364; doi:10.2196/jmir.3734)
Supplement: Supplementary file 1 [file jmir_v17i4e94_app1.pdf]

AD HOC QUESTIONNAIRE

1. Which of these fatty acids is polyunsaturated ?
   a. Palmitic acid
   b. Butyric acid
   c. Linoleic acid
   d. All of the above
   e. None of the above

2. One calorie is approximately equal to
   a. 4.2 Joule
   b. 0.42 Joule
   c. 96.8 Joule
   d. 9.68 Joule
   e. 968 Joule

3. Vitamin B1 deficiency can cause
   a. Fractures
   b. Beriberi
   c. Influenza
   d. Sjögren’s syndrome
   e. Creutzfeldt-Jakob disease

4. Cobalamin is also known as
   a. Vitamin B12
   b. Vitamin A
   c. Pepsin
   d. Vitamin E
   e. Cellulose

5. To calculate the body mass index, it is necessary
   a. to divide the height in centimeters by the square of the body mass in kilos
   b. to multiply the height in meters by the cube of the body mass in kilos
   c. to subtract the height in meters from the body mass in kilos
   d. to divide the body mass in kilos by the square of the height in meters
   e. to multiply the height in centimeters by the body mass in kilos

6. The energy intake from proteins introduced through a balanced diet should be
   a. 10-15% of the daily energy intake
   b. 20-25% of the daily energy intake
   c. 25-30% of the daily energy intake
   d. 30-40% of the daily energy intake
   e. 40-45% of the daily energy intake

7. 100 grams of butter contain approximately
   a. 356 Kcal
   b. 256 Kcal
   c. 1652 Kcal
   d. 320 Kcal
   e. 717 Kcal

8. Which of these amino acids is essential ?
   a. Tryptophan
   b. Alanine

c. Asparagine
d. Proline
e. Serine

9. The energy intake from carbohydrates introduced through a balanced diet should be
   a. 55-75% of the daily energy intake
   b. 35-55% of the daily energy intake
   c. 15-30% of the daily energy intake
   d. At least 80% of the daily energy intake
   e. At least 15% of the daily energy intake

10. The energy intake from fats introduced through a balanced diet should be
    a. as little as possible of the daily energy intake
    b. 10-15% of the daily energy intake
    c. 3-5% of the daily energy intake
    d. 6-8% of the daily energy intake
    e. 15-30% of the daily energy intake

Correct answers are **in bold**
